# Quality assessment of large language model–generated prior authorization letters in nephrology

**DOI:** 10.3389/fdgth.2026.1767648

**Published:** 2026-03-03

**Authors:** Noppawit Aiumtrakul, Charat Thongprayoon, Chutawat Kookanok, Methavee Poochanasri, Kitinan Phichedwanichskul, Wisit Cheungpasitporn

**Affiliations:** 1 Division of Nephrology and Hypertension, Department of Medicine, Mayo Clinic, Rochester, MN, United States; 2 Department of Medicine, One Brooklyn Health, Interfaith Medical Center, Brooklyn, NY, United States; 3 Department of Medicine, Bhumibol Adulyadej Hospital, Bangkok, Thailand; 4 Phramongkutklao Hospital, Bangkok, Thailand

**Keywords:** artificial intelligence, ChatGPT-5, large language models, nephrology, prior authorization

## Abstract

**Background:**

Prior authorization (PA) is a major source of administrative burden, treatment delay, and clinician burnout. Artificial intelligence (AI), particularly large language models (LLMs), is increasingly used to assist with clinical documentation, yet its reliability for payer-facing administrative tasks remains uncertain.

**Objective:**

To evaluate the quality of PA letters drafted by ChatGPT-5 for commonly used medications requiring PA in nephrology. Quality was evaluated based on correctness and strength of clinical reasoning.

**Methods:**

We created a single standardized prompt and applied it across 29 nephrology scenarios to generate PA letters. Each PA letter was reviewed against four criteria: 1) absence of false statements or hallucinations, 2) correctness of ICD-10 coding, 3) presence and validity of citations, and 4) clinical reasoning, rated on a 4-point Likert scale (illogical, weak, adequate and strong). FDA drug labels, KDIGO guidelines and related randomized controlled trials were used as reference standards.

**Results:**

Out of 29 letters, one letter (3.5%) contained false statements mentioning an irrelevant clinical trial. The ICD-10 diagnosis code was correct in 23 letters (79.3%), most errors were related to chronic kidney disease (CKD) staging or internal diagnostic inconsistencies. 27 letters (93.1%) cited valid references, with one letter citing an incorrect trial and another one citing a correct KDIGO guideline with inaccessible link. Twenty-six letters (89.7%) demonstrated strong clinical reasoning, supported by guideline-oriented or FDA label–aligned justification. The remaining 3 letters were rated as adequate reasoning. The main areas for improvement involved citing relevant references and emphasizing special considerations, for example Risk Evaluation and Mitigation Strategy (REMS) compliance for eculizumab.

**Conclusions:**

ChatGPT-5 can generate clinically coherent PA drafts for nephrology medications, but limitations in coding precision and citation reliability persist. With appropriate oversight, AI-assisted documentation may reduce administrative burden while maintaining safety and accuracy.

## Introduction

An American Medical Association (AMA) survey reported that 93% of physicians believe prior authorization (PA) has a negative impact on patient care, and 95% reported an association with professional burnout (1). Twenty four percent of survey respondents reported serious consequences of PA, including permanent impairment, hospitalization, or death (1). PA is a utilization management process used by health insurers to determine whether a prescribed medication, procedure, or service meets predefined criteria for coverage before reimbursement is approved (2, 3). In practice, PA typically requires clinicians to submit detailed documentation outlining the clinical indication, accurate diagnostic coding, prior treatment history, justification of medical necessity, and supporting evidence aligned with clinical guidelines or regulatory labeling (4–6). Physicians spend an estimated 12–13 h per week submitting approximately 39 PA requests (1, 7).

Physicians report that PA delays or insurer hurdles lead patients to discontinue care, with up to 78% noting that they have seen treatment abandonment in their own practice (8). Delays created by PA often leave physicians with less time for direct patient care and add to the administrative load required to keep treatment plans moving. These interruptions can affect patients' health and also add pressure on clinicians, contributing to frustration, reduced efficiency, and higher operating costs, as noted in recent reports from the AMA and the Healthcare Business Management Association (HBMA) (7, 8). Although detailed data for individual specialties remain limited, emerging evidence shows that PA requirements create similar challenges across a broad range of clinical conditions. A recent systematic review conducted by Johns Hopkins University that examined 25 primary studies from the United States found consistent reports of treatment delays, disease exacerbations, avoidable hospitalizations, prolonged inpatient stays, and worse survival outcomes, particularly in cancer care (9). These effects were seen across oncology, cardiology, behavioral health, pediatrics, rheumatology, and infectious diseases, suggesting that the burden of PA is not confined to any single area of practice (9).

Conceptually, PA and AI-assisted documentation can be understood through three complementary theoretical perspectives (2, 10–12). Health services research frames PA as a utilization management mechanism intended to promote evidence-based and cost-conscious care, while simultaneously introducing administrative burden and potential barriers to timely treatment when documentation quality is suboptimal (12). Theories of human-AI collaboration view large language models as assistive tools designed to augment clinician workflows rather than replace clinical judgment, particularly for documentation-intensive tasks. In parallel, trust and reliability frameworks for clinical decision support systems emphasize that adoption depends on consistent performance across core domains such as factual accuracy, transparency of reasoning, and appropriate use of supporting evidence (13–15). Together, these perspectives underscore the importance of systematically evaluating AI-generated documentation in high-stakes administrative contexts.

Given the complexity and high stakes of PA submissions, the quality of documentation, including the accuracy of clinical reasoning, diagnostic coding, and supporting references, is critical to successful approval (2–6). In recent years, there has been increasing interest in the use of artificial intelligence (AI), particularly large language models (LLMs), to support documentation-intensive tasks in medicine (16–19). These tools are already being applied to generate a range of clinical communications, including faxed submissions to health plans, letters to patients, referral notes, and inter-provider correspondence among them (20). For example, roughly one-quarter of pediatricians report using such systems to help prepare letters, request PAs, or support patient and family education (21).

Despite growing interest in clinical applications, the performance of LLMs in real medical settings remains inconsistent. A recent systematic review found that ChatGPT answered medical questions correctly only about half the time, with an overall accuracy of 56% (95% CI, 51%–60%) (22). In nephrology, LLMs accuracy varied widely, with dietary potassium and phosphorus classification ranging from 66%–100% across models (23). In a separate citation study, correct references accounted for only 3%–38% of outputs across different LLMs (24). These inconsistencies underscore the need for caution when applying LLM-generated text to high-stakes administrative tasks such as PA submissions, where factual errors, incorrect coding, or inappropriate citations may directly affect patient access to care.

ChatGPT, developed by OpenAI, is a large language model designed to generate human like text and assist with information retrieval and writing tasks (25). It is now commonly used for summarizing articles (26), drafting academic writing (27), and organizing complex information (28, 29). Several published studies, including prior work by our group, have evaluated LLM performance in medicine and nephrology, focusing on general medical question answering, specialty-specific knowledge, educational use cases, and the reliability of generated citations (17, 30–37). However, these studies have largely emphasized feasibility, general accuracy, or informational tasks rather than structured evaluation of payer-facing administrative documents.

Given the substantial administrative burden associated with PA and the rapid adoption of AI-based drafting tools, it is timely to examine whether LLMs can meaningfully reduce workload without compromising accuracy, clinical reasoning, or safety. The differentiating factor of the present study is its focus on a high-stakes, payer-facing administrative use case rather than general medical question answering or narrative documentation. The objective of this study was to systematically evaluate the quality of prior authorization letters generated by ChatGPT-5 for commonly encountered nephrology scenarios, with specific assessment of factual accuracy, ICD-10 coding correctness, citation validity, and the strength of clinical reasoning using a standardized, task-specific framework.

## Materials and methods

We developed 29 standardized nephrology clinical scenarios involving medications commonly requiring PA. Each scenario included a diagnosis coded using the International Classification of Diseases, 10th Revision (ICD-10), with medication indications supported by FDA-approved labeling (38), KDIGO guideline (39), and major randomized trials. PA letters were generated using ChatGPT-5 (OpenAI) accessed via the web-based interface, using default model settings (no user-specified temperature, top-p, or token limits) (Supplementary Material 1). No system-level messages, custom instructions, retrieval tools, plugins, or external reference materials were provided beyond the standardized prompt. All outputs were captured verbatim and were not edited or post-processed prior to evaluation.

### LLM setup and prompting

A single standardized prompt was used across all cases to ensure consistency. The prompt instructed the model to draft a professional PA letter as a board-certified nephrologist, clearly state the indication and regimen, assign the most specific ICD-10 code(s), justify medical necessity, and include at least one supporting reference with a full URL. The prompt and scenario-specific information were submitted together as a single input. All letters were generated on September 4, 2025:

“You are a board-certified Nephrologist writing a prior authorization (PA) letter to health plan medical reviewers in a professional tone.

Task: Draft a ≤ 350-word PA letter for the scenario below.

Requirements:

1. Clear statement of the indication and requested regimen/dose.
2. Diagnosis with ICD-10 code(s): choose the most specific and appropriate code(s)
3. Clinical reasoning: why this medicine is medically necessary for this patient
4. References section with at least one clinical guideline or high-quality source. Provide hyperlinks as full URLs.”

The prompt and clinical scenario were entered together in sequence. The standardized prompt was placed first, followed by the scenario details for that specific case, and the combined text was submitted as a single input for the model to generate the PA letter.

### Evaluation

**Figure 1:**
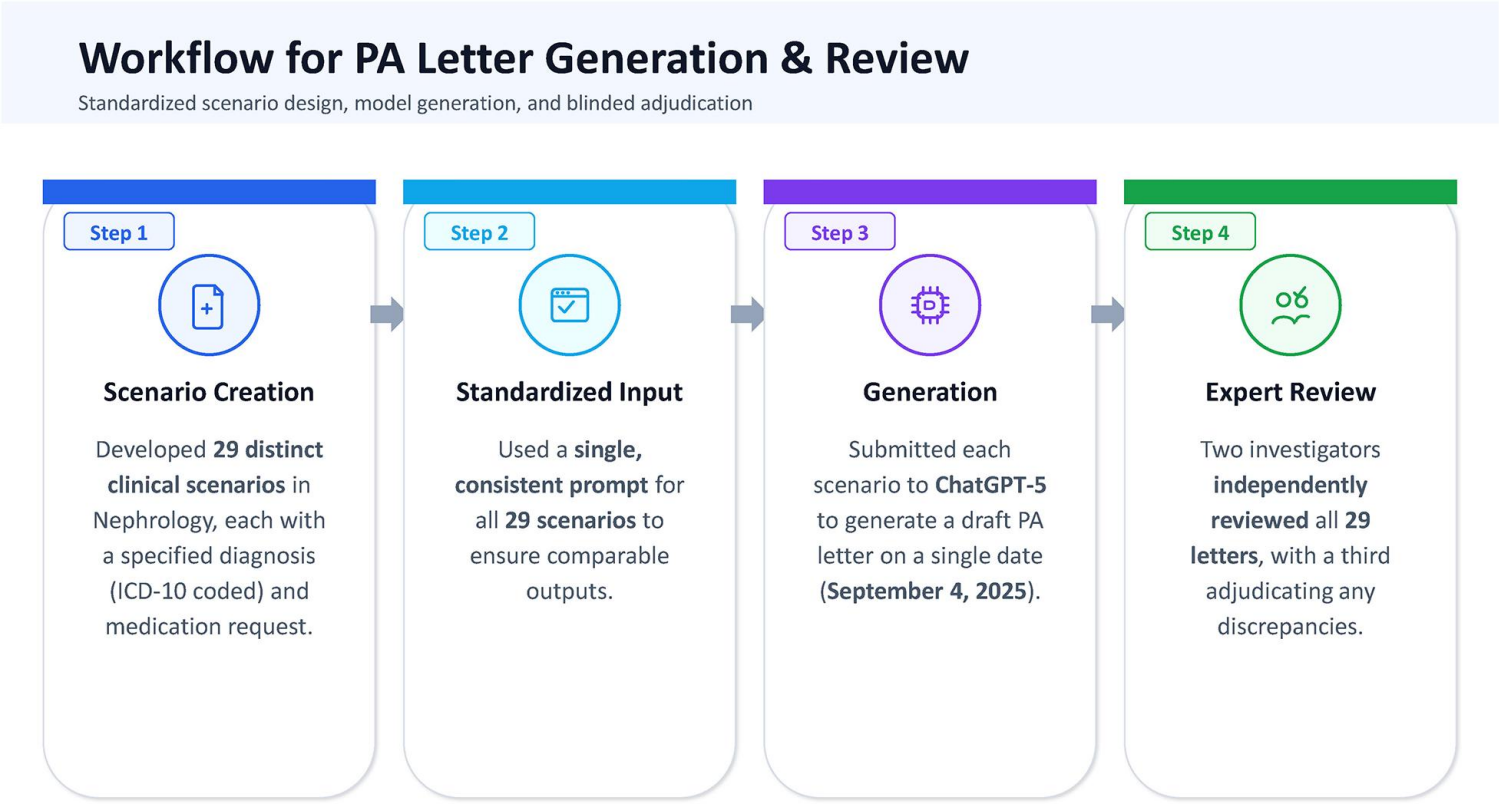
Workflow for the generation and evaluation of ChatGPT-5–produced prior authorization letters in nephrology.

Each generated letter was then reviewed using four criteria: (1) the presence or absence of false statements, (2) correct use of ICD-10 coding, (3) the accuracy of any cited references, and (4) the strength of the clinical reasoning. Clinical reasoning was scored on a four-level Likert scale. “Illogical” (score 1) was assigned when explanations were inconsistent with the patient information or the drug label. “Weak” (score 2) reflected minimal or incomplete justification. “Adequate” (score 3) indicated a plausible rationale that covered key points without depth. “Strong” (score 4) was given when the reasoning integrated patient-specific factors, guideline-supported arguments, and appropriate safety considerations. Two investigators (N.A. and C.K.) independently reviewed all 29 letters and recorded their assessments separately. The results were then compared, and any discrepancies were resolved through adjudication by a third investigator (W.C.). The presence of a false statement, accurate ICD-10 coding, and valid references were analyzed as binary variables (yes/no). Clinical reasoning was categorized as “strong” (score 4) and all other scores. An overview of the workflow appears in Figure 1. This study was conducted and reported in accordance with the TRIPOD-LLM guideline for transparent reporting of studies evaluating large language models in healthcare (40).

## Results

**Figure 2:**
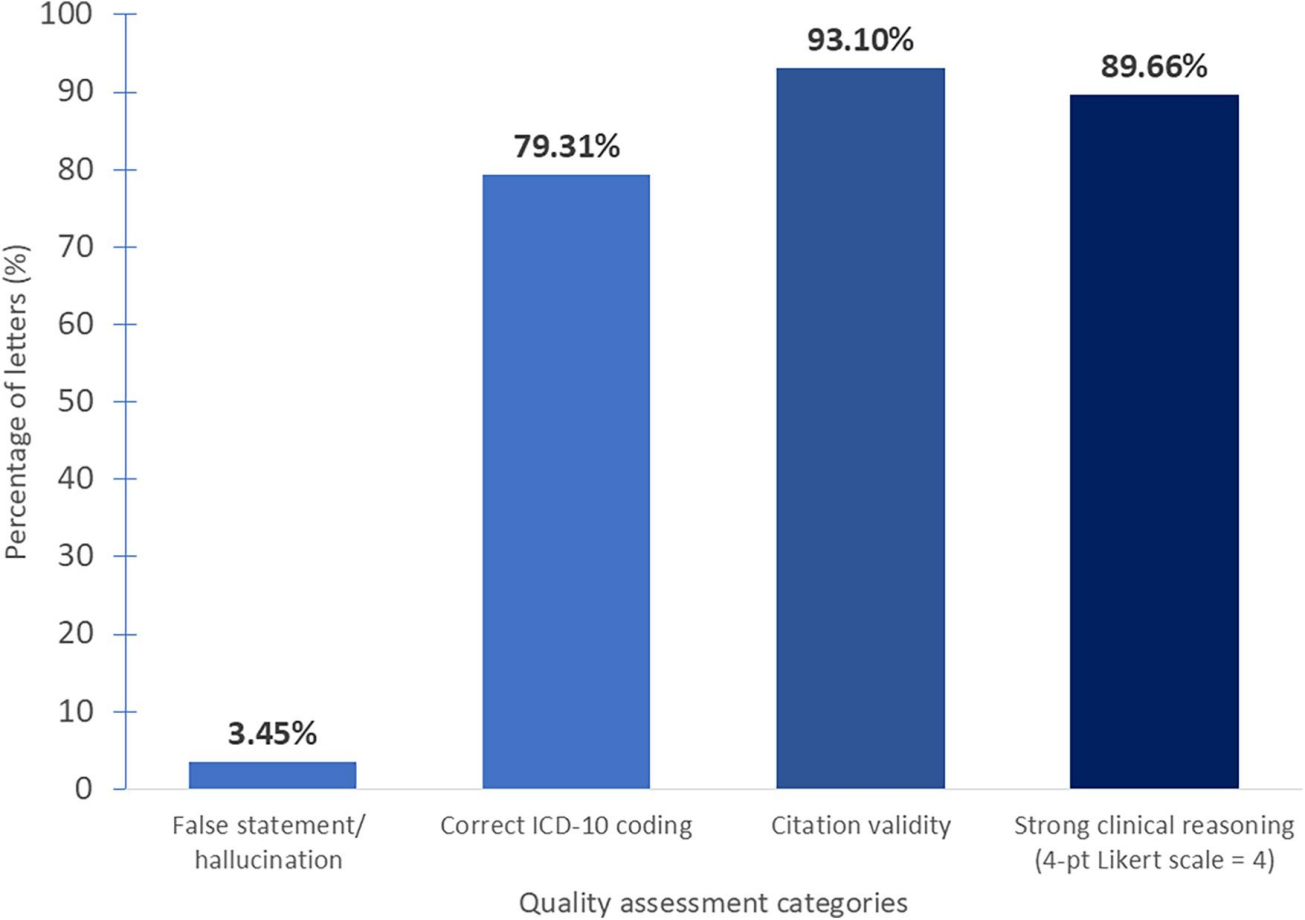
Performance of AI-generated prior authorization letters across four evaluation domains.

Among the 29 ChatGPT-5-generated PA letters reviewed, most met the basic expectations for accuracy, diagnostic coding, reference use, and clinical justification (Figure 2). Only 1 letter (3.5%) contained a false statement. ICD-10 coding was correct in 23 letters (79.3%), and 27 letters (93.1%) used valid citations. Clinical reasoning was the strongest domain, with 26 letters (89.7%) rated as strong and the remaining 3 letters (10.3%) still rated as adequate. Errors were generally narrow in scope and concentrated in predictable areas such as chronic kidney disease (CKD) staging, citation accuracy, and omission of key safety considerations. A more detailed examination of each domain is described below.

### False statements

A single letter (3.5%) included a factual error. In the velphoro scenario, the text referenced the INNO2VATE trial (41), which evaluates vadadustat for anemia and has no relevance to phosphate binders. This error resulted from citation of an unrelated clinical trial and therefore represents both a factual inaccuracy and a reference mismatch, rather than an incorrect description of the medication's indication or mechanism of action. This was the only instance we found in which a clearly unrelated clinical trial was cited (Supplementary Figure S1).

### ICD-10 coding issues

ICD-10 inaccuracies were found in 6 letters (20.7%). The most frequent issue involved chronic kidney disease staging. Several scenarios, particularly those involving sodium–glucose cotransporter 2 (SGLT2) inhibitors or disease-modifying therapies such as tolvaptan, sparsentan, and iptacopan, assigned stage 3b (N18.32) despite clinical information consistent with stage 3a (N18.31), with estimated GFR values clustered around 46 to 50 mL/min/1.73 m^2^ (42). Another miscoding appeared in the rituximab letter for granulomatosis with polyangiitis (43). The narrative clearly described renal involvement, yet the letter listed both a code for GPA without kidney involvement and the code for GPA with kidney involvement, creating an inconsistent and confusing diagnostic description (Supplementary Figure S2).

### Reference mismatches

Reference accuracy was high overall, with 27 of 29 letters (93.1%) citing appropriate sources. Two letters required correction. One occurred in the nedosiran scenario, where the model cited data from lumasiran rather than the PHYOX clinical program supporting nedosiran (44, 45). Another letter linked to a KDIGO guideline but provided a non-functional URL (Supplementary Figure S3–S4).

### Clinical reasoning

Strong clinical reasoning was observed in 26 letters (89.7%), all of which provided patient-specific justification aligned with guidelines or FDA-approved labeling. Three letters (10.3%) were rated as adequate rather than strong. The tenapanor letter appropriately addressed indication but omitted key points about gastrointestinal tolerability and monitoring (46). In the nedosiran scenario, the rationale was plausible but lacked depth and was paired with the citation mismatch noted earlier. The eculizumab letter offered a clear justification for treating atypical HUS after transplant but failed to mention essential safety measures, including meningococcal vaccination and prophylaxis per FDA-approved labeling (47). These omissions represent incomplete reasoning rather than incorrect conclusions (Supplementary Figure S4–S6).

## Discussion

This study provides an early look at how ChatGPT-5 performs when asked to generate PA letters for commonly used nephrology medications. Overall, the model produced PA letters that were mostly accurate, appropriately structured, and supported by strong clinical reasoning. Most submissions were free of factual errors, the majority used correct ICD-10 coding, and almost all cited reasonable sources. These findings suggest that, at baseline, the tool can create letters that resemble what clinicians routinely prepare in practice.

The pattern of errors is instructive. The single false statement identified in the sample was not subtle. Citing the INNO2VATE vadadustat trial (41) in support of a phosphate binder illustrates how confidently the model may pull in unrelated information. Although infrequent, these errors underscore the importance of verifying sources to ensure accuracy and consistency in clinical documentation. A similar issue was seen in the nedosiran scenario in which data from the lumasiran program were used instead of the PHYOX trials (44, 45) that form the evidence base for nedosiran. These errors may seem small but can weaken the credibility of a PA submission, especially when reviewers scrutinize supporting literature.

ICD-10 coding represented another area where lapses were more common (48). The most frequent mistake involved staging CKD (42). Several scenarios with eGFR values around 45 to 50 mL/min per 1.73 m^2^ were labeled as stage 3b rather than the correct stage 3a based on the provided eGFR values. In the rituximab scenario, both a code for GPA with renal involvement and a code without renal involvement were listed together, creating a confusing and internally inconsistent diagnostic picture. These errors did not alter the clinical intent of the letters but did reduce the overall precision of diagnostic coding. For payers who rely on correct coding to determine benefit coverage, this level of inconsistency can introduce unnecessary friction.

Despite these shortcomings, clinical reasoning was the model's strongest domain. Nearly ninety percent of letters offered a well-constructed explanation grounded in patient-specific details and aligned with guideline or FDA-approved labeling criteria. The remaining letters fell short not because the indications were incorrect but because important considerations were omitted. These included gastrointestinal tolerability for tenapanor (46), discussion of safety monitoring for nedosiran, and meningococcal vaccination or prophylaxis for eculizumab (47). These are elements a human author would typically include automatically because they are tied to risk-mitigation strategies or boxed warnings. Their absence is a reminder that LLM-generated text may overlook details that clinicians regard as routine.

Several established quantitative metrics have been proposed to evaluate LLM performance, including accuracy scores, factual consistency measures, and text similarity benchmarks (35, 49, 50). However, most of these metrics were developed for general natural language processing tasks or medical question answering and do not adequately capture the task-specific requirements of PA letters (35, 49–51). In the PA context, clinically meaningful errors often relate to diagnostic coding precision, appropriateness of cited evidence, or completeness of payer-facing clinical justification, domains that are not well reflected by generic LLM performance metrics (35, 49, 50). Accordingly, we intentionally adopted a domain-specific, clinician-centered evaluation framework that prioritizes attributes directly relevant to PA review and approval. This approach is intended to complement, rather than replace, existing LLM benchmarking strategies by emphasizing practical reliability in a high-stakes administrative setting (15, 35, 49, 50, 52).

Our findings highlight a concern but encouraging picture. ChatGPT-5 reliably captures the overall framework of PA justification and articulates it clearly in many cases. At the same time, it can overlook coding nuances, substitute incorrect trial data, or omit safety considerations that are critical for payer review. These limitations are manageable if the tool is used to support rather than replace clinician judgment. With thoughtful clinician supervision, AI-generated drafts may reduce the administrative time required to prepare PA letters, but they cannot yet be relied on without thorough review. As health systems consider adopting such tools, attention to validation, error-checking workflows, and clinician sign-off will be essential to ensure safe and accurate use.

Several study limitations should be emphasized. The evaluation was based on a fixed set of 29 standardized and relatively straightforward nephrology scenarios, which do not capture the full complexity, ambiguity, or longitudinal context of real-world PA requests. All letters were generated using a single model version at a single time point, and performance may vary across model updates or alternative architectures. In addition, the study focused on document-level quality metrics and did not assess payer-facing outcomes such as approval rates, turnaround times, or the need for appeals.

Future research should extend this work to more complex and less structured clinical scenarios, evaluate performance across multiple LLMs and model versions, and examine real-world payer responses to AI-assisted PA submissions. Integrating electronic health record data, along with automated checks for diagnostic coding accuracy, drug–evidence alignment, and safety requirements, may further improve reliability (53, 54). Prospective studies measuring administrative efficiency, clinician workload, and downstream payer outcomes will be critical to defining the appropriate role of LLMs in supporting PA workflows.

## Conclusion

ChatGPT-5 generated PA letter drafts that were generally accurate and well structured, with acceptable clinical reasoning in most scenarios. The errors mainly involved coding, reference selection, and incomplete safety discussions. Our findings highlight the need for careful review before use. With appropriate oversight, LLM-generated drafts may help reduce administrative burden, but they are not yet reliable enough to be used without clinician verification.

## Data Availability

The original contributions presented in the study are included in the article/Supplementary Material, further inquiries can be directed to the corresponding author.
