# Supplementary material for: Quality assessment of large language model–generated prior authorization letters in nephrology
Source: Front Digit Health. 2026 Mar 3;8:1767648. doi: 10.3389/fdgth.2026.1767648 (PMC12992280; doi:10.3389/fdgth.2026.1767648)
Supplement: Supplementary file 1 [file Datasheet1.pdf]

Supplementary Materials 1

Listed below are the 29 nephrology clinical scenarios evaluated in this study.

1. Secondary hyperPTH in Dialysis dependent ESRD
   Calcimimetic: Cinacalcet (Sensipar)

Scenario: A 65-year-old male with end-stage renal disease (ESRD) on hemodialysis for 3 years with a known secondary hyperparathyroidism presents with worsening bone pain, muscle weakness and pruritus. The patient has consistently elevated serum intact parathyroid hormone (iPTH) at 800 pg/mL and phosphate levels at 6.8 mg/dL despite the use of vitamin D analogs and phosphate binders. His recent serum calcium level was 8.9 mg/dL. Cinacalcet is planned to be initiated orally at 30 mg once daily to control elevated iPTH levels and manage symptoms associated with secondary hyperparathyroidism in ESRD.

2. Hyperphosphatemia in CKD
   Phosphate Absorption Inhibitors: Tenapanor

Scenario: A 72-year-old female with ESRD on hemodialysis for 3 years has consistently high serum phosphate levels (6.8 mg/dL) despite the use of phosphate binders. She is planned to be started on tenapanor 30 mg twice daily to reduce phosphate absorption in combination with her current therapy.

3. Secondary hyperparathyroidism in advanced CKD
   Vitamin D Analogue: Paricalcitol

Scenario: A 60-year-old male with CKD stage 4 is found to have secondary hyperparathyroidism with an elevated parathyroid hormone (PTH) level of 400 pg/mL and normal serum calcium. Paricalcitol is planned to be initiated orally 1 mcg once daily to reduce PTH levels and manage secondary hyperparathyroidism.

4. Anemia due to CKD
   Erythropoiesis-Stimulating Agent (ESA): Darbepoetin Alfa

Scenario: A 50-year-old female with CKD stage 4 and normocytic anemia presents with hemoglobin of 9.5 g/dL and symptoms of fatigue and dyspnea on exertion. Her iron stores are adequate, but erythropoietin levels are low. She is planned to be started on darbepoetin alfa 25 mcg once every 2 weeks subcutaneously to stimulate erythropoiesis and improve her hemoglobin levels.

5. Anemia due to CKD
   HIF Stabilizer: Daprodustat

Scenario: A 48-year-old male with end-stage renal disease (ESRD) on hemodialysis presents with hemoglobin of 8.5 g/dL and symptoms of fatigue and dyspnea on exertion, who has previously failed to respond to erythropoiesis-stimulating agents (ESAs), is planned to be initiated on daprodustat 4 mg orally once daily to increase endogenous erythropoietin production via hypoxia-inducible factor (HIF) stabilization.

6. Chronic hyperkalemia in advanced CKD
   Potassium Binders: Patiromer Sorbitex Calcium

Scenario: A 55-year-old male with CKD stage 5 not on dialysis and poorly controlled diabetes presents with persistent hyperkalemia (serum potassium approximately 6.0 mEq/L) despite dietary modifications and the discontinuation of medications associated with hyperkalemia. He is planned to be prescribed patiromer 8.4 mg once daily orally to manage hyperkalemia.

7. CKD secondary to Diabetic Nephropathy
   Sodium-Glucose Cotransporter 2 (SGLT2) Inhibitor: Empagliflozin

Scenario: A 62-year-old female with type 2 diabetes, diabetic retinopathy and CKD stage 3 with a recent eGFR of 46 mL/min/1.73 m², presents with progressively increasing albuminuria (400 mg/g) despite receiving the maximum dose of losartan. She is planned to be started on empagliflozin 10 mg once daily, taken orally, to reduce the progression of CKD.

8. CKD secondary to Diabetic Nephropathy
   Mineralocorticoid (Aldosterone) Receptor Antagonist: Finerenone

Scenario: A 62-year-old female with type 2 diabetes, diabetic retinopathy and CKD stage 3 with a recent eGFR of 40 mL/min/1.73 m², presents with progressively increasing albuminuria (400 mg/g) despite receiving the maximum dose of losartan and empagliflozin. Her recent potassium and morning cortisol level were within normal range. She is planned to be started on finerenone 10 mg once daily, taken orally, to reduce the progression of CKD and cardiovascular events associated with diabetic nephropathy.

9. Autosomal Dominant Polycystic Kidney Disease (ADPKD)
   Vasopressin V2 Receptor Antagonist: Tolvaptan

Scenario: A 45-year-old male with a family history of ADPKD presents with rapidly progressive worsening renal function (eGFR 50 mL/min/1.73 m²) and bilateral enlarged kidneys with multiple cysts on ultrasound. His recent liver function was within normal range and urine osmolality was 280 mOsm/kg. Tolvaptan is planned to be prescribed initially at 60 mg/day in divided doses to slow the progression of cyst growth and preserve kidney function.

10. Primary IgA Nephropathy
    Endothelin type A receptor and angiotensin II type 1 receptor antagonist: Sparsentan

Scenario: A 35-year-old female with biopsy-proven IgA nephropathy presents with persistent proteinuria (2 g/day) despite receiving maximal RAAS blockade for 3 months. Her recent eGFR was 50 mL/min/1.73 m², liver function was within normal range and urine pregnancy test was negative. The patient is currently on effective contraception. She is planned to be started on sparsentan 200 mg once daily to reduce proteinuria and possibly slow the progression of IgA nephropathy.

11. Granulomatosis with Polyangiitis

CD20-directed cytolytic antibody: Rituximab

Scenario: A 45-year-old male presented with a 3-week onset of productive cough, nasal congestion, hematuria, oliguria and worsening shortness of breath. Initial workup showed rapidly worsening renal function with recent serum creatinine at 3.4 mg/d, active urinary sediment and subnephrotic-range proteinuria. Anti-neutrophil cytoplasmic antibodies was positive for c-ANCA (anti-PR3). Granulomatosis with Polyangiitis was suspected, and a

kidney biopsy confirms severe active ANCA-associated glomerulonephritis. He is planned to receive Rituximab IV 375 mg/m² once weekly for 4 doses as induction therapy, in combination with high-dose glucocorticoids. HBV screening is negative, LFTs are normal, and his vaccinations are up to date.

12. Severe active anti-neutrophil cytoplasmic autoantibody (ANCA)-associated vasculitis with RPGN
Complement 5a receptor (C5aR) antagonist: TAVNEOS® (avacopan)
Scenario: A 58-year-old female presents with hematuria, oliguria, fatigue, and worsening dyspnea over a few weeks. Labs reveal a serum creatinine of 3.8 mg/dL, active urinary sediment, and ANCA positivity (c-ANCA/PR3). Kidney biopsy confirms necrotizing crescentic glomerulonephritis, consistent with severe active ANCA-associated vasculitis with RPGN. She is planned to start TAVNEOS® (avacopan) 30 mg twice daily, in combination with glucocorticoids. HBV screening is negative, LFTs are normal, and and her vaccinations are up to date.

13. Primary IgA Nephropathy
Complement Factor B Inhibitor: FABHALTA® (iptacopan)
Scenario: A 40-year-old male with biopsy-proven primary IgA nephropathy presents with rapid renal progression, evidenced by a urine protein-to-creatinine ratio (UPCR) of 2.0 g/g. His recent serum creatinine is rising from 1.0 to 2.3 mg/dL during the past few months. The patient is afebrile, and vaccinations, especially for encapsulated bacteria, are up to date. He is planned to be started on FABHALTA® (iptacopan) 200 mg twice daily orally to slow the progression of his IgA nephropathy and reduce proteinuria.

14. Primary IgA Nephropathy
Systemic Corticosteroid: TARPEYO® (budesonide)
Scenario: A 37-year-old female with **biopsy-proven primary IgA nephropathy** presents with a **urine protein-to-creatinine ratio (UPCR) of 1 g/g** despite 3 months of maximal RAAS inhibition therapy. She is considered at high risk for chronic kidney disease progression. As an alternative immunosuppressive therapy, she is planned to start **TARPEYO® (budesonide)** oral capsules at 16 mg once daily in the morning for 9 months to reduce proteinuria and potentially slow disease progression.

15. Hyperphosphatemia in CKD
Phosphate binders: Sevelamer
Scenario: A 72-year-old female with nondialysis-dependent CKD stage V has consistently high serum phosphate levels (6.8 mg/dL) despite dietary phosphate restriction. She is planned to be started on Sevelamer 800 mg three times daily to reduce phosphate absorption.

16. Hyperphosphatemia in CKD
Phosphate binders: Sucroferric oxyhydroxide (Velphoro)

Scenario: A 72-year-old female with ESRD on hemodialysis for 3 years has consistently high serum phosphate levels (6.8 mg/dL) despite dietary phosphate restriction. She is planned to be started on sucroferric oxyhydroxide (Velphoro) 500 mg 3 times daily to reduce phosphate absorption.

17. Hyperphosphatemia in CKD
Phosphate binders: Ferric citrate
Scenario: A 72-year-old female with ESRD on hemodialysis for 3 years has consistently high serum phosphate levels (6.8 mg/dL) despite dietary phosphate restriction. She is planned to be started on Ferric citrate 2000 mg 3 times daily to reduce phosphate absorption.

18. Secondary hyperPTH in Dialysis dependent ESRD
Calcimimetics: Etelcalcetide
Scenario: A 65-year-old male with end-stage renal disease (ESRD) on hemodialysis for 3 years with a known secondary hyperparathyroidism presents with worsening bone pain, muscle weakness and pruritus. The patient has consistently elevated serum intact parathyroid hormone (iPTH) at 800 pg/mL and phosphate levels at 6.8 mg/dL despite the use of vitamin D analogs and phosphate binders. His recent serum calcium level was 8.9 mg/dL. Etelcalcetide is planned to be initiated intravenously at 5 mg 3 times per week at the end of dialysis to control elevated iPTH levels and manage symptoms associated with secondary hyperparathyroidism in ESRD.

19. Anemia due to CKD
IV iron: INJECTAFER® (ferric carboxymaltose injection)
Scenario: A 50-year-old female with CKD stage 4 and new-onset normocytic anemia presents with a hemoglobin of 8.0 g/dL and symptoms of fatigue and dyspnea on exertion. Her lab work reveals a Mean Corpuscular Volume (MCV) of 70 fL, Red Cell Distribution Width (RDW) of 16%, Serum Iron of 20 µg/dL, Total Iron Binding Capacity (TIBC) of 650 µg/dL, Transferrin Saturation of 4%, Ferritin of 8 ng/mL, and a Reticulocyte Count of 0.8%. These findings are consistent with iron deficiency anemia. She is planned to receive ferric carboxymaltose (INJECTAFER®) 750 mg intravenously, administered in two doses separated by at least 7 days, to improve her iron stores and hemoglobin levels.

20. Anemia due to CKD
Erythropoiesis-Stimulating Agent (ESA): Methoxy polyethylene glycol-epoetin beta (Mircera)
Scenario: A 50-year-old female with CKD stage 4 and normocytic anemia presents with hemoglobin of 9.5 g/dL and symptoms of fatigue and dyspnea on exertion. Her iron stores are adequate, but erythropoietin levels are low. She is planned to be started on Methoxy polyethylene glycol-epoetin beta (Mircera) 40 mcg once every 2 weeks subcutaneously to stimulate erythropoiesis and improve her hemoglobin levels.

21. Anemia due to CKD
HIF Stabilizer: vadadustat
Scenario: A 48-year-old male with ESRD on hemodialysis 3 times weekly and anemia of CKD presents with hemoglobin of 8.5 g/dL and symptoms of fatigue and dyspnea on exertion,

who has previously failed to respond to erythropoiesis-stimulating agents (ESAs), is planned to be initiated on vadadustat 300 mg orally once daily to increase endogenous erythropoietin production.

22. CKD secondary to Diabetic Nephropathy
Sodium-glucose cotransporter 2 (SGLT2) inhibitor: FARXIGA (dapagliflozin)
Scenario: A 62-year-old female with type 2 diabetes, diabetic retinopathy and CKD stage 3 with a recent eGFR of 46 mL/min/1.73 m^2, presents with progressively increasing albuminuria (400 mg/g) despite receiving the maximum dose of losartan. She is planned to be started on dapagliflozin 10 mg once daily, taken orally, to reduce the progression of CKD.

23. CKD secondary to Diabetic Nephropathy
SGLT2i: Canagliflozin
Scenario: A 62-year-old female with type 2 diabetes, diabetic retinopathy and CKD stage 3 with a recent eGFR of 46 mL/min/1.73 m^2, presents with progressively increasing albuminuria (400 mg/g) despite receiving the maximum dose of losartan. She is planned to be started on Canagliflozin 100 mg once daily, taken orally, to reduce the progression of CKD.

24. Chronic hyperkalemia in advanced CKD
Potassium binders: LOKELMA® (sodium zirconium cyclosilicate)
Scenario: A 55-year-old male with CKD stage 5 not on dialysis and poorly controlled diabetes presents with persistent hyperkalemia (serum potassium approximately 5.7 mEq/L) despite dietary modifications and the discontinuation of medications associated with hyperkalemia. He is planned to be prescribed sodium zirconium cyclosilicate 10 g 3 times daily for up to 48 hours, followed by 10 g once daily to manage hyperkalemia.

25. Anemia due to CKD
Erythropoiesis-Stimulating Agent (ESA): Epogen® (epoetin alfa)
Scenario: A 50-year-old female with CKD stage 4 and normocytic anemia presents with hemoglobin of 9.5 g/dL and symptoms of fatigue and dyspnea on exertion. Her iron studies were within normal range, but erythropoietin level was low. She is planned to be started on epoetin alfa 4000 units once weekly subcutaneously to stimulate erythropoiesis and improve her hemoglobin levels.

26. Kidney Stone From Primary Hyperoxaluria type 1
A double-stranded siRNA: RIVFLOZA (nedosiran)
Scenario: A 50-year-old male with primary hyperoxaluria type 1 presents with kidney stones and progressive decline in kidney function. His most recent estimated glomerular filtration rate (eGFR) was 40 mL/min/1.73 m^2. He is scheduled to start treatment with RIVFLOZA (nedosiran) at a dose of 160 mg once monthly to reduce hyperoxaluria and preserve kidney function.
27.Paroxysmal Nocturnal Hemoglobinuria/ IgA nephropathy (expanded)

Selective oral factor B inhibitor: Fabhalta (ravulizumab-cwvz)
Scenario: A 36-year-old female with a history of paroxysmal nocturnal hemoglobinuria (PNH) presents with fatigue, abdominal pain, and recurrent episodes of dark urine. Laboratory tests reveal hemoglobin levels of 8.2 g/dL, elevated lactate dehydrogenase (LDH), and low haptoglobin. He is schedule to start Fabhalta 200 mg orally twice daily to reduce hemolysis and alleviate symptoms.

28. Atypical Hemolytic Uremic Syndrome
Soliris (Eculizumab)
Scenario: Scenario: A 59-year-old female with a past medical history of obesity and T2DM was admitted to the hospital due to atypical HUS. Her initial lab work was significant for thrombocytopenia (90, 000/uL), with Hb of 9.8 g/dL. In addition, she had an acute kidney injury with a creatinine of 5.75 mg/dL and blood urea nitrogen of 56 mg/dL. Her ADAMTS13 came back as 52% (normal >60%), and stool analysis was negative for Shiga toxins. Her complement levels were low with C3 75 mg/dL [83-193 mg/dL], C4 8 mg/dL [15-57 mg/dL], and low total complement (35.4 U/mL) [38.7-89.9 U/mL]. Ultomiris is planned to be prescribed a loading dose of 3,000 mg IV, followed by 3,600 mg IV for maintenance dose 2 weeks later, then 3,600 mg once every 8 weeks thereafter.

29. Atypical Hemolytic Uremic Syndrome
Ultomiris (Ravukuzumab-cwvz)
Scenario: A 37-year-old female was hospitalized with atypical HUS She has a history of T1DM and also had pancreatic and renal transplantation. Laboratory results revealed Hb of 10.7gm/dl, platelets at 125,000/µl, a creatinine of 0.5, and a peripheral blood smear revealed occasional schistocytes with reduced platelets. Complement studies revealed a C3 level of 57 (normal range: 80 to 160 mg/dL) and a C4 level of 16.4 (normal range: 16 to 48 mg/dL). ADAMTS 13 was positive at 58%. Eculizumab is planned to be prescribed 900 mg weekly for the first 4 weeks, followed by 1200 mg for the fifth dose 1 week later, then 1200 mg every 2 weeks thereafter to inhibit complement-mediated thrombotic microangiopathy
